# Editorial: The role of genes and network pharmacology in new drug discovery

**DOI:** 10.3389/fgene.2025.1578094

**Published:** 2025-03-05

**Authors:** Jingxin Mao

**Affiliations:** ^1^ Chongqing Medical and Pharmaceutical College, Chongqing, China; ^2^ College of Pharmaceutical Sciences, Southwest University, Chongqing, China

**Keywords:** network pharmacology, gene, new drug discovery, molecular biology, genome sequencing

In the ever-evolving field of new drug development, genetics and network pharmacology have emerged as two pivotal elements that are revolutionizing the traditional paradigm of drug research and development (Hopkins, 2008). Historically, new drug development has relied heavily on broader and more empirical approaches. However, with advances in genetic science and network pharmacology, this field is undergoing a profound transformation.

Genes, as the basic units of inheritance, are critical to understanding the underlying molecular mechanisms of disease. In the past, the understanding of disease was limited to superficial symptoms. The advent of high-throughput sequencing technology has enabled the decoding of the entire human genome, opening the door for researchers to the microscopic world and providing a vast amount of information on various disease-related gene variations (Nizamani et al., 2023). This invaluable knowledge allows researchers to identify potential drug targets with unprecedented precision. In cancer research, for instance, the discovery of specific oncogenes such as breast cancer susceptibility gene (BRCA) 1 and BRCA2 has directly led to the development of targeted therapies such as poly (ADP-ribose) polymerase protein (PARP) inhibitors (Hobbs et al., 2021). These drugs can precisely target cancer cells carrying BRCA mutations, leading to more significant therapeutic effects and drastically reduced side effects compared to traditional chemotherapy, thereby greatly improving the treatment experience and quality of life for cancer patients.

On the other hand, network pharmacology takes a holistic view of complex biological systems. It recognizes that diseases are not caused by a single gene or protein but rather by perturbations in intricate molecular networks. By thoroughly analyzing the interactions between genes, proteins, and small molecules, network pharmacology aims to identify multitarget drugs that can regulate multiple nodes in disease-related networks (Mao et al., 2024). This approach is particularly important for complex diseases such as neurodegenerative disorders and metabolic syndromes, which are often caused by multiple deregulated signaling pathways. For example, in Alzheimer’s disease research, network-based drug discovery strategies are exploring drugs that can simultaneously target amyloid-beta (*β)* aggregation, tau phosphorylation, and neuroinflammatory pathways, potentially bringing new therapeutic hope to Alzheimer’s patients (Mayo et al., 2024).

However, the integration of genetics and network pharmacology in the development of new drugs presents numerous challenges. One major obstacle is the complexity of data analysis. Large-scale genomic data generated by high-throughput experiments, along with complex network-related data, require advanced bioinformatics and computational tools for accurate interpretation (Lin, 2024). Furthermore, new drug targets identified through these methods still require time-consuming and resource-intensive preclinical and clinical validation. From laboratory research to animal experiments and human clinical trials, each step demands substantial time, manpower, and funding, and issues at any stage can hinder the entire research and development process (Hodos et al., 2016).

Therefore, the title of this Research Topic “*The role of genes and network pharmacology in new drug discovery*” was carried out which aims to better provide the academic forefront of computational methods for biomedical research in pharmacology and medicine in healthcare big data. A total of four original research articles were collected from well-known authors in the relevant field. Lu et al. found that through various molecular and computer simulation experiments the pituitary tumor transforming gene (PTTG) gene family (PTTG1, PTTG2, and PTTG3P) is continuously upregulated in osteosarcoma (OS) cell lines and has the potential to serve as a diagnostic biomarker. The related cytosine-phosphate-guanine (CpG) islands exhibit significant hypomethylation, gene mutations are rare, and functional assays confirm their carcinogenic effects. The authors also determined that calcitriol is the most suitable drug for PTTG gene therapy of OS, which opens up new avenues for understanding the pathogenesis of OS and developing targeted therapies. Wang and Mao investigated the pharmacological mechanism of *β*-sitosterol in the treatment of rheumatoid arthritis. Through network pharmacology and molecular docking experiments, they demonstrated that *β*-sitosterol can effectively bind to six core targets, significantly inhibit the excessive proliferation of MH7A cells, and regulate the expression of related genes. It may be possible to inhibit rheumatoid arthritis by regulating the forkhead box (Fox)O and phosphatidylinositide 3-kinase (PI3K)/protein kinase B (AKT) signaling pathways. Zhang et al. identified prognostic genes related to lung adenocarcinoma (LUAD) through univariate analysis and used machine learning to construct a pre-screened key gene small nuclear ribonucleoprotein polypeptide A (SNRPA) for *in vitro* experiments on LUAD cell lines. This study demonstrates the prognostic value and clinical application of nucleotide metabolism in LUAD. Prognostic features constructed based on nucleotide metabolism-related genes can accurately predict patient prognosis and guide immunotherapy for LUAD. Hossain et al. conducted a comprehensive bioinformatics analysis of the factors involved in cyclin-dependent kinases regulatory subunit (CKS) 1B in LUAD and lung squamous cell carcinoma (LUSC) and explored its potential role as a biomarker for early detection and treatment of lung cancer. The study demonstrated the immunotherapeutic characteristics and prognostic significance of CKS1B in cancer progression.

Taken together, genetics and network pharmacology have opened up new avenues for new drug development. By integrating genetic information and network-based analytical power, researchers can develop more effective and personalized drugs. Overcoming current challenges in data analysis and target validation is critical to realizing the full potential of these innovative methods in the pharmaceutical industry. Only by continuously overcoming technical bottlenecks and strengthening multidisciplinary collaboration can we accelerate the development of new drugs and bring more treatment options and hope for recovery to patients worldwide.
